# A double blind controlled trial comparing three treatment modalities for dentin hypersensitivity

**DOI:** 10.4317/medoral.17594

**Published:** 2011-12-06

**Authors:** Nilam Brahmbhatt, Neeta Bhavsar, Vishal Sahayata, Aneesha Acharya, Payal Kshatriya

**Affiliations:** 1 Senior Lecturer, Department of Periodontology, Government Dental College and Hospital, Ahmedabad. Gujarat; 2 Professor And Head, Department of Periodontology, Government Dental College and Hospital, Ahmedabad. Gujarat; 3 Senior Lecturer, Department of Periodontology, Faculty of Dental Science, Dharamsinh Desai University Nadiad. Gujarat; 4 Senior Lecturer, Department of Periodontology, Dr D Y Patil Dental College and Hospital, Pimpri, Pune; 5 Graduate Student, Department of Public Health Dentistry, University of Medicine and Dentistry of New Jersey

## Abstract

Aim: This randomized, double blind, split mouth study was aimed to compare three dentin desensitizing treatment modalities. Methods: Two hundred sixty teeth of 25 patients; each having at least 2 hypersensitive teeth in each quadrant, were included. Teeth were randomized to 4 groups: Group A treated with 2% NaF solution, Group B received GLUMA®; an aqueous solution of Hydroxy-Ethyl-Methacrylate and Glutarldehyde, (HEMA-G), Group C received iontophoresis with distilled water (placebo) and Group D was treated with NaF-iontophoresis. Pain response was evaluated on a visual analogue scale (VAS), by using tactile, air blast and cold-water stimuli at 0-day, 15-day, 1-month and 3-months interval. Results: All treatments were effective in reducing dentinal hypersensitivity significantly, Group D and Group B were more effective than Group A and Group C at all time intervals. Group D and Group B were equally effective in reducing dentinal hypersensitivity at 15-day and 1-month interval but Group D was more effective at 3-months. Conclusion: All treatment modalities were more effective in reducing hypersensitivity than placebo. 2% NaF-iontophoresis and HEMA-G were more effective than 2% NaF local application at all time intervals. But at 3-months, 2% NaF-iontophoresis was more effective than HEMA-G, while placebo produced no significant effect in reduction of hypersensitivity.

** Key words:**Hypersensitivity, desensitisation, iontophoresis, dentin adhesive, sodium fluoride.

## Introduction

Dentinal hypersensitivity is common clinical condition and an age-old complaint, presenting problems to both the patient and dentist and is reported to be relatively widely prevalent (1). Although several hypotheses have been advanced to explain how external stimuli may influence the nerve fibers, the most widely accepted is the hydrodynamic theory (2,3).

Extensive research has been done on the treatment of hypersensitive dentine, but no treatment is accepted universally. Compliant with the hydrodynamic theory, prevention and /or relief of the pain can be accomplished by sealing the outer end of the dentinal tubules, coagulation of dentinal protoplasm, blocking the pulpal end of the dentinal tubules by formation of the secondary dentin or by anesthetizing nerve endings of the pulp (4,5). Various therapeutics rationales have led to different clinically proven treatment modalities. These include nerve depolarization (6); as occurs with topical potassium nitrate, protein binding and calcium compound deposition within tubules; as with ‘tooth mousse’ or Casein phosphopeptide – Amorphous Calcium Phosphate (7), increased peritubuar mineral deposition; as with topical glucorticoid therapy (8) and photobiomodulation leading to tubular occlusion that Laser desensitization is based upon (9).

Studies have shown that topical application of NaF exerts a desensitizing effect on exposed dentin, but its effect is transient (10,11). Iontophoresis of NaF has gained some popularity, a technique in which fluoride can be transferred under electrical pressure deep into the dentinal tubules, has been utilized (12,13). It possibly causes calcium fluoride precipitation, which may decrease fluid movement induced by stimuli, reducing dentin hypersensitivity.

Sealing the dentinal tubules with a bonding agent or adhesive material has also been suggested to create long lasting blockage of dentine hypersensitivity (14). One such product is an aqueous solution of hydroxyl-ethyl-methacrylate and glutaraldehyde (HEMA-G), and a strong desensitizing effect of this system on dentin hypersensitivity has been reported (15,16). It blocks the dentinal tubules by coagulation of the dentinal fluid proteins within the tubules, thereby counteracting the hydrodynamic mechanism of dentinal hypersensitivity.

The comparative efficacies of various desensitizing treatments are still unclear in spite of availability of multiple treatment modalities, hence the present study was planned to evaluate and compare the efficacy of 2% sodium fluoride solution with and without iontophoresis with a commercially available adhesive desensitizing agent.

## Material and Methods

This three-month clinical trial included 260 teeth of 25 individuals of both sexes, age ranging from 20 to 50 years selected from Department of Periodontology, Government Dental College and Hospital, Ahmedabad; who demonstrated tooth hypersensitivity on buccal surface of the teeth to heat, cold and mechanical stimuli, at least two teeth in each quadrant. The Institutional Review Board and ethical committee approved the study protocol, and written and verbal informed consent was obtained from all study participants.

Exclusion criteria were; history of current desensitizing therapy, cracked tooth, chipped teeth, defective restorations, deep periodontal pockets or a tender tooth in same quadrant, orthodontic appliances, bridge work, dentures, deep dental caries or large restoration showing pulpal response, periodontal surgery within last 6 months, any chronic systemic disease or cardiac pacemakers.

-Study design

A split mouth study design was adopted; having advantages of same pain perception, oral hygiene habits, dietary habits and psychosomatic factors. Each subject’s oral cavity was divided into 4 quadrants; different drugs were applied in each. In order to avoid bias on the part of the investigators and the patient, a double blind technique was used, where neither the scorer of the pain who examined the patients or nor the patient himself was aware of the solution’s name. Before the hypersensitivity treatment, phase I therapy was completed.

Sensitivity/ pain response was assessed by using the Numerical 0-10 Visual Analogue Scale (VAS) (17). Pre treatment sensitivity was evaluated on the by the first investigator. After the test stimuli were applied at baseline, the teeth rated five or more on VAS for any of the two tests were selected for the study. All the teeth to be treated were isolated by cotton rolls and dried by air. Different drugs were randomly applied to each quadrant according to the choice of the second investigator. The first investigator then evaluated the post treatment VAS response by applying test stimuli at 0-day immediately after treatment, 15 days, 1-month and 3-months.

-Test stimuli 

The tooth number and the location of hypersensitive area were recorded and subjects thus screened were examined for baseline sensitivity. For all test stimuli, a 0-10 numerical rating VAS scale was adopted and the patient was asked to provide a numerical VAS rating with 0 indicating ‘no pain’ and 10 indicating ‘intolerably severe pain’.

1) Tactile test (Mechanical method): A sharp dental explorer (17\23) was passed lightly across the affected area, perpendicular to long axis of tooth. The test is repeated three times before the score was recorded.

2) Air blast test: Air blast from dental syringe at 60-pound/inches 2 pressure was directed on to the tooth for 1 second from a distance of 10 mm.

3) Thermal (Water test).

Cold water test –Using a plastic syringe and a starting temperature of 20°C, water was flown on to the sensitive surface for maximum of 3 seconds. If the response was negative the teeth was retreated with a temperature of 10°C or 0°

In any case, when the discomfort became intolerable the stimulus was immediately removed. Throughout the study, the test stimuli were applied in the same order, with minimum 5-minute interval between the applications of different stimuli.

-Procedure for drug application

Drug-A

The 2% sodium fluoride solution was prepared freshly each time by dissolving 200 mg of medical grade sodium fluoride powder (DNS Fine Chemicals, Mumbai, India) in 10 ml of distilled water (Nirmal Prime Health Care, Ahmedabad, India) in a sterile plastic bottle. A cotton pledglet soaked in 2% NaF solution was placed on the exposed cervical dentin of the tooth for period of three minutes and the solution was dropped on to the cotton pledged each minute.

Drug-B

GLUMA® solution (Heraeus Kulzer, South Bend, USA) was dispended from a 5 ml plastic bottle containing 5% gluteraldehyde, 35% Hydroxy-Ethyl-Methacrylate and Glutarldehyde (HEMA-G) and purified water. A drop of desensitizer was applied using a cotton applicator and left for 30 sec. and then dried with stream of air until the fluid film disappeared and the surface was no longer shiny.

Drug-C 

Iontophoresis was performed by using a commercially available instrument; Desensitron II (Parkell Inc, Farmingdale, USA). The forked end of an autoclaved probe was inserted into the socket at the front of the power unit. A cotton pledget was inserted into a disposable white plastic applicator tube and pushed into the tube with the probe until it was exposed at the other end. The cotton pledged was wetted sufficiently with several drops of distilled water. Holding the device in one ungloved hand and touching the patient with the other ungloved hand completed the circuit. The current was gradually increased to the selected level, a maximum of 0.5mA, and this current was applied for 2 minutes per tooth, comprising a dosage of 1mA per minute.

Drug-D 

The tooth and iontophoresis instrument were prepared similarly. The cotton pledged was wetted sufficiently with several drops of freshly prepared 2% sodium fluoride solution. The circuit was completed, and the current was applied for 2 minutes per tooth. Some extremely sensitive patients complained of slight discomfort, in those cases; the current was reduced below patient’s threshold by turning the current knob counterclockwise. However, treatment time was increased proportionally.

-Statistical Analysis

Uncoding of data was done after 3-months by breaking the code of the drug. Percentage of reduction in hypersensitivity from pre-treatment level was determined by tabulating the numerical change in VAS score at any given point and expressed as a percentage value of the pretreatment score.

All data obtained was appropriately tabulated and a statistical analysis was performed to assess and compare the levels of significance (P value < 0.05) for mean percentage reduction of VAS scores by applying Paired T test for intragroup comparison and Student’s T test for inter-group comparisons.

## Results

A total of 265 teeth were treated in 25 patients aged between 20 to 50 years. These included 64 teeth treated with Drug A, 65 teeth treated with Drug B, 70 treated with Drug C and 65 treated with Drug D, respectively.

Drug A or 2% sodium fluoride induced mean reduction of hypersensitivity VAS scores for all three test stimuli were 43%, 41%, 40% immediately after application on 0-day; which increased to 52%, 55%, 45% on the 15th day; and 35%, 34%, 36% after 1-month. After 3-months, the mean reductions in hypersensitivity were 24%, 22%, 22% respectively (Table I). Thus, 2% sodium fluoride showed significant reduction (p<0.05) in hypersensitivity from baseline on 0-day and 15th day for all stimuli, with both tactile and air blast test showing highly significant (p<0.01) reduction on day 15. At 1-month reduction in sensitivity remained significant for all except for cold stimulus. At 3-months both air blast and cold water test were not significant and only tactile test showed significant reduction from baseline.

Reduction in hypersensitivity with Drug-B or GLUMA® (Table I) was highly significant (p<0.01) on 0-day &and 15th day for all stimuli, and at 1-month &and 3-months for tactile stimulus, while remaining at significant (p<0.05) level for air blast and cold water test at the 1 and 3 month intervals. Reduction scores for all stimuli were 82%, 80%, &and 76% at 0-day; increased to 92%, 93% &and 88% at 15th day; and 73%, 75%, &and 69% at 1-month. On evaluation at 3-months, the reduction in hypersensitivity was 55%, 52%, &and 50% respectively.

Drug-C or placebo iontophoresis with distilled water (Table 1) caused significant (p<0.05) reduction in hypersensitivity scores compared to baseline (for tactile and air blast test) on 0-day. Reduction in hypersensitivity for all stimuli were 28%, 29%, 22% immediately after application at 0-day. At 15-day, the reduction scores were; 20%, 19%, 15%, which was non significant for tactile test. At 3-months no (0%) reduction in hypersensitivity was noted for all stimuli.

Drug-D or ionotophoresis with 2% sodium fluoride ( Table 1) caused highly significant (p<0.01) reduction in hypersensitivity on 0 day, 15th day and 1 month for all three stimuli. At 3 months, tactile and air blast test remained at highly significant levels while cold-water test dropped to a significant level (p<0.05). The reduction in hypersensitivity at 0-day were 85%, 89%, 84% respec-tively for all stimuli, while on 15th day the scores increased to 95%, 96%, 91%. At 1-month a drop occurred to 82%, 85%, 78%, which changed to 73%, 74%, 68% at 3-months.

**Table 1:**
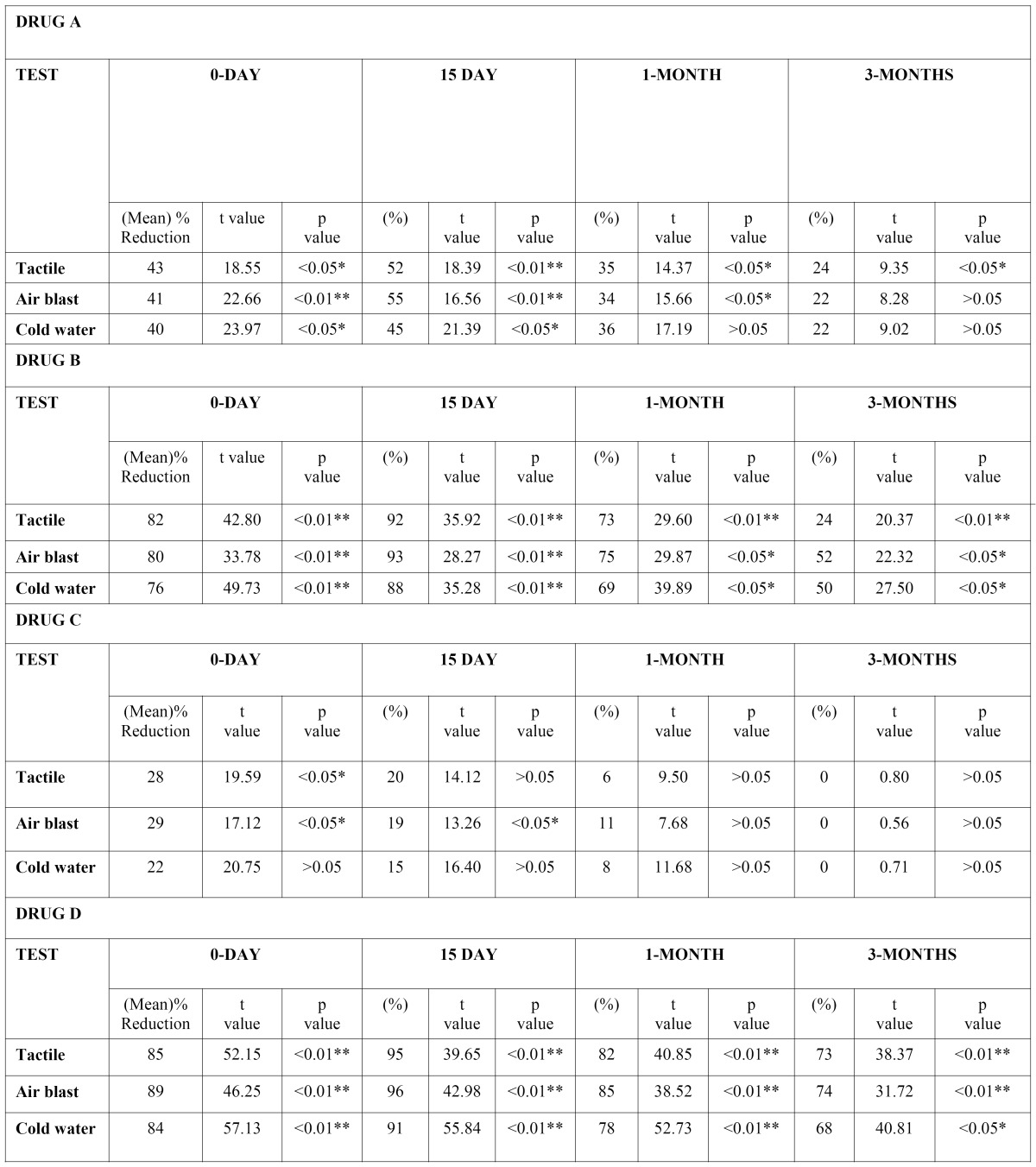
Intra-group comparison of percentage of hypersensitivity reduction from baseline (1).

Group comparisons ( Table 2) indicate that with regard to immediate hypersensitivity reduction; Drug A or sodium fluoride without iontophoresis was significantly less effective than both Drug B (GLUMA®) and Drug D (sodium fluoride with iontophoresis) and as compared to placebo (Drug C), it showed significantly better results only with the air blast test, (p<0.05). However, at 15 days and 1 month, Drug A became significantly better than placebo with both tactile and cold-water tests. No significant difference was noted between Drug A and placebo at 3 months. Drug A remained less significantly effective at all other times intervals compared to both Drugs B and D.

**Table 2:**
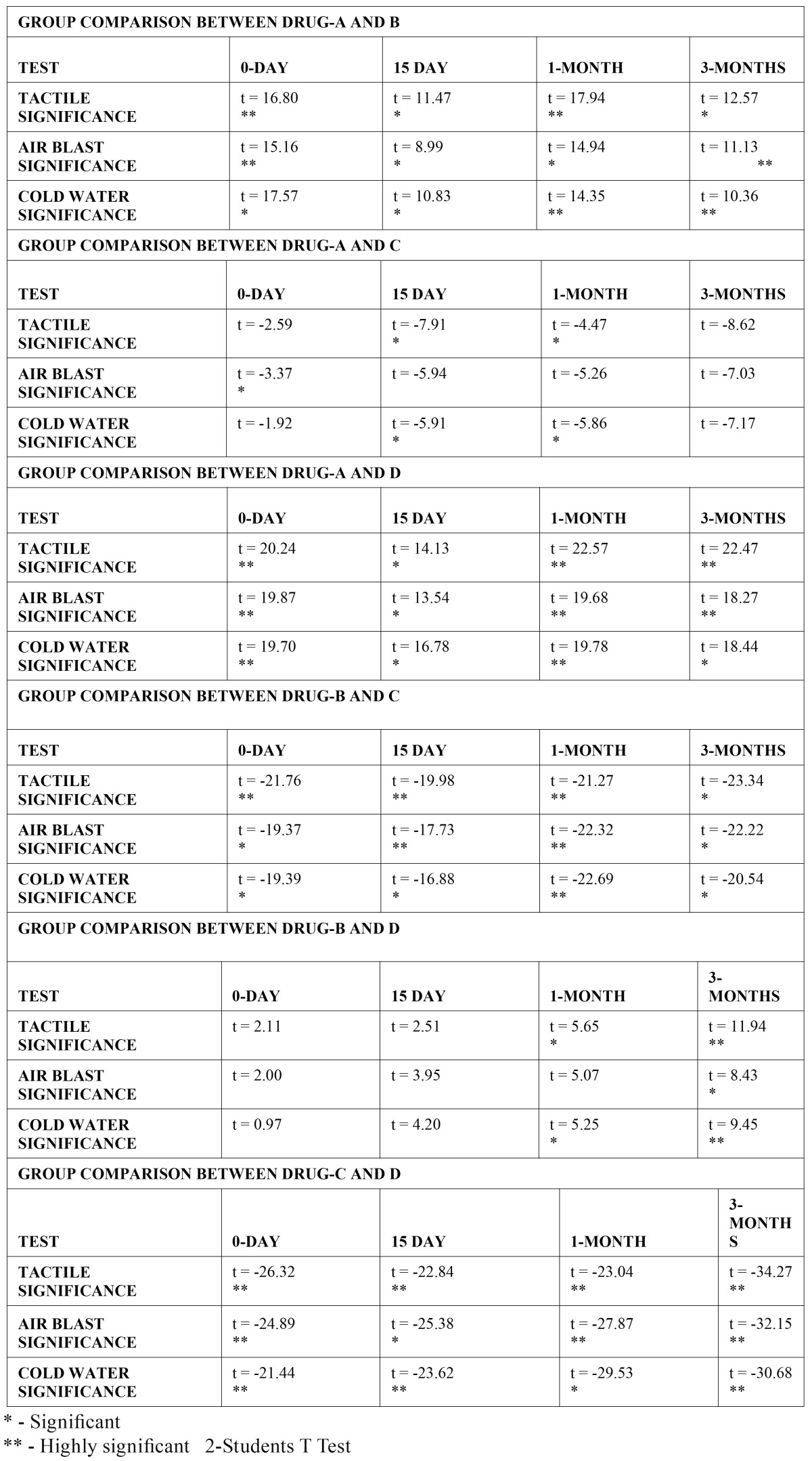
Group comparisons between drugs (2).

Comparing Drugs B and D, non-significant differences were noted at 0 day and 15 day. However, at 1 month, there was significantly more hypersensitivity reduction by Drug D with tactile and cold-water tests. At 3 months, this difference was also significant with air-blast test, while being highly significant with tactile and cold-water tests. Both drugs B and D were significantly more effective than placebo at all intervals. Although, at 3 months the comparison of placebo with Drug D remained highly significant (p<0.01), while that of placebo with Drug B was significant (p<0.05), using all three tests.

## Discussion

Dentinal hypersensitivity is a very distinct and annoying clinical problem. Affected patients avoid and can no longer enjoy hot cold, chilled, acidic or sweet liquid and food18. Selection of the correct treatment modality is based on the premise of proven clinical efficacy both in terms of magnitude and duration of desensitizing effect. Lack of proven universal acceptance of any one such treatment creates the need for a comparative analysis of most commonly accepted desensitizing treatments. The present study was carried out using double blind, randomized, parallel group and split mouth design (19,20).

Sensitive teeth were exposed to tactile, air blast, and cold-water stimulus simultaneously and in the same manner as it is described before (21,22). Scoring for hypersensitivity was done with numerical rating Visual Analogue Scale. The validity and reliability of VAS for measuring both experimental &and clinical pain had been demonstrated (17). The 0-10 numerical rating VAS scale has been shown to be an efficacious alternative to the continuous VAS, while being simpler in application and patient comprehension (23). Other pain quantifying assessment modes include the intensity verbal descriptor or unpleasantness verbal indicator, but are more subject to inaccuracy than VAS. Although force-probes or scratchometer have been widely used, they were not used in our study because these are contraindicated in evaluating treatment by adhesive restorative materials i.e. HEMA-G (24).

Sodium fluoride purportedly blocks dentinal tubules through the precipitation of calcium fluoride crystals. Topical application is sometimes transient and incomplete (11). Therefore iontophoresis was envisioned as a method of electrically transporting fluoride ions into the dentinal tubules (12,25). Burke and Malik (26) suggested sealing of the tubules or impregnating them with some bonding resin or adhesive material. This action was attributed to gluteraldehyde, which is a biological fixative that may denature the protein in the dentinal fluid, thereby occluding the dentinal tubule (27). HEMA-G, being water soluble, might promote deep penetration of glutarldehyde into the tubules leading to formation of peripheral intrinsic barrier consisting of multiple thin septa within its lumen (16).

Reduction of sensitivity with sodium fluoride application did not show immediate results as also observed by Tal et al (28). Sodium fluoride application caused a modest reduction of hypersensitivity. Precipitation of CaF2 crystals causing reduction of the functional radius of dentinal tubules occurs in 1 to 4 weeks after sodium fluoride application (29-31). The results also show reduction in efficiency at 3-months for all stimuli that may be attributed to a fairly rapid loss of the occluding layer of CaF2 as it is diluted by saliva (32). This may explain why topical application of NaF has limited effectiveness in reducing sensitivity in long-term basis.

The significant effect of the dentin adhesive desensitizer GLUMA® is attributed to glutaraldehyde, which occludes dentinal tubules by protein precipitation (27,33,34). A highly significant immediate relief effect was as noted before (33,34). However, there was a reduction in the effectiveness of the product at 3-months, probably as a result of loss or wear of the occluding layer (35).

Iontophoresis with distilled water, as a control treatment demonstrated significant reduction of hypersensitivity scores at 0 and 15 days. These results are likely to be due to placebo effect of the instrument, similar to those reported by McBride et al (21).

Our findings at 0-day indicated that sodium fluoride iontophoresis provides immediate effect from dentinal hypersensitivity as noted previously36, possibly because of microprecipitaion of calcium fluoride within the dentinal tubules (25). Lefkowitz et a and Burdilkl (37) proposed that application of current results in formation of reparative dentin and dead tracks that prevent the passage of stimuli from exposed dentin to the pulp. We noted the best results at 15th day to 1-month in this study, which may denote that adequate amount of reparative dentin is formed after 1 to 3 weeks. Results of therapy at 1-month and 3-months show the long-term effect of 2% sodium fluoride iontophoresis, possibly consistent with the hypothesis that electrically driven fluoride ions react with calcium in the hydroxyapatite to form fluorapatite, which blocks the dentinal tubules (38). The magnitude of results did not reach 100% and could reflect the requirement of more current or current loss through adjacent soft tissue (39). Similar to that observed for GLUMA®, the reduction in the effectiveness of the product at the end of study is likely to be due to loss or wear of the occluding layer (40).

On comparative analysis, GLUMA® showed better immediate effect as compared to topical 2% sodium fluoride at all time intervals. This is likely to be due to intradentinal sealing observed with dentin adhesives.16. Conversely; sodium fluoride takes time to form calcium fluoride crystals. Comparing sodium fluoride with and without iontophoresis, 2% sodium fluoride had no immediate effect on reduction of hypersensitivity, while 2% sodium fluoride with iontophoresis was demonstrated to have an immediate post treatment effect (40).

Comparing the effects of the most effective treatments GLUMA® and 2% sodium fluoride iontophoresis; while similar efficacies are noted at 0, 15 day time intervals, from 1 month toward 3 month intervals iontophoresis resulted in significantly better results. Olusile et al, in a short term study reported a better immediate response to GLUMA® compared to fluoride iontophoresis, but noted a better response at 7 days with iontophoresis 41; although we did not find GLUMA® to be superior at 0 day, our l and 3 month findings validate those of previous studies denoting that iontophoresis provides better long term relief (35,41). In in -vivo situations, the effectiveness of adhesives can be compromised over time as the seal between the adhesive and the dentin surface breaks down, consequently dentine sensitivity may reoccur (42) and is possibly the explanation for the decreased long-range efficacy that we noted.

The highly subjective nature of the condition makes it extremely difficult to evaluate dentine hypersensitivity objectively (43). The potential limitation of studying treatment responses to dentinal hypersensivity is the subjective assessment of response that obviously lacks standardized measurability, (44), although we utilized a split mouth study design to limit the influence of inter subject response variability. Studies incorporating larger sample sizes are essential to further validate our findings.

## Conclusions

The results of our study indicated all three agents, 2% NaF local application, 2% NaF iontophoresis and HEMA-G (GLUMA®) desensitizer were effective in decreasing sensitivity as compared to placebo for all stimuli at different time intervals. 2% NaF iontophoresis and HEMA-G were equally effective on all test stimuli at different time intervals. Placebo or iontophoresis with distilled water did not show significant improvement in hypersensitivity at any stage of study. Topical 2% sodium fluoride without iontophoresis, although does not provide any immediate effect but cause modest relief of hypersensitivity after 15th day. Its effect gradually wears, so it is not a long-term reliable drug. GLUMA® and 2% sodium fluoride with iontophoresis both cause immediate relief in hypersensitivity. The efficacy of both these drugs is somewhat similar up to duration of 1-month. However at 3-months interval, 2% sodium fluoride iontophoresis was comparatively more efficient, indicating a longer lasting effect.
